# Annual Conference of the GMA, Hamburg, Germany, 2014

**DOI:** 10.3205/zma000958

**Published:** 2015-05-13

**Authors:** Wolfgang Hampe, Olaf Kuhnigk

**Affiliations:** 1 University Medical Center Hamburg-Eppendorf, Department of Biochemistry and Molecular Cell Biology, Hamburg, Germany; 2 University Medical Center Hamburg-Eppendorf, Office of the Vice Dean of Education, Medical Faculty of Hamburg University, Hamburg, Germany

## Report

**Figure 1:**
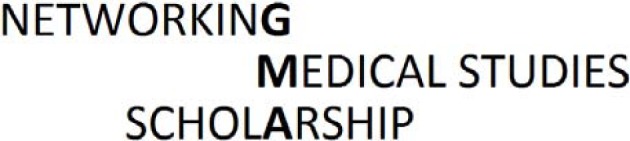


The motto (see Figure 1 (Fig. 1)) these topics was the focus of the annual conference of the GMA, held from September 25-27, 2014, in Hamburg, Germany. Almost 700 participants met in the “Campus Lehre”, the main teaching facility of the University Medical Center Hamburg-Eppendorf, to learn and discuss in 118 lectures, 21 workshops, 18 GMA committee meetings, and with 200 posters what is new in scientific teaching. Beside the interesting lectures and poster presentations, the excellent keynote speakers provided the audience with food for thought: Jonathan Silverman from Cambridge talked about teaching in clinical communication, Geoff Norman from McMaster distinguished between effective and less effective means to improve medical education at universities. Hans-Jochen Heinze, chairman of the Medicine Committee of the German Council of Science and Humanities, committedly presented the council’s current recommendations for the advancement of medical education in Germany. Olaf von dem Knesebeck (UKE) and Victor Oubaid of the German Aerospace Center completed the programme with thoughts on how to educate socially competent medical doctors and on the possibility of transferring knowledge gained in flight security and pilot selection to medical education and student admission tests.

**Figure 2:**
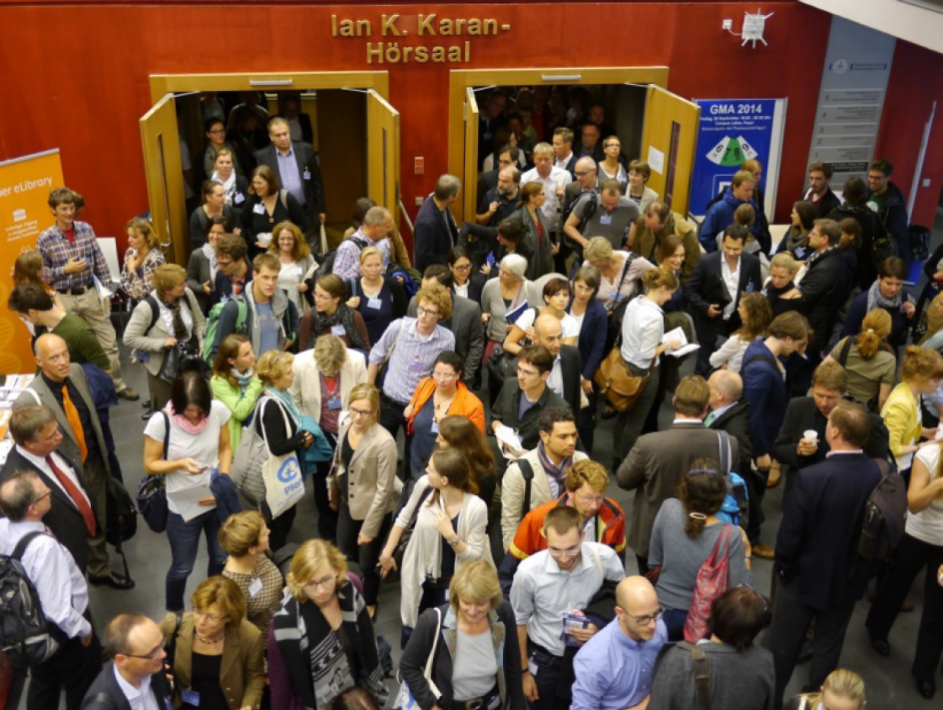
Foyer of the Campus Lehre (photo: Birgit Henkel, UKE)

Fortunately, the GMA annual conferences have been growing continuously over the past years, and during the meeting in Hamburg, the 1,000^th^ society member was received! It proved to be a major logistic challenge to arrange all contributions evaluated by the more than 40 reviewers reasonably between Thursday afternoon and Saturday lunchtime. The generous spaciousness of the site of the meeting (see Figure 2 (Fig. 2)) allowed the temporal separation of the various types of contributions such as poster tours and short lectures, though. Thus, we were able to ensure the participants’ full attention for all formats. This was only possible by placing the committee meetings and workshops outside of the actual congress time. In case that the interest in the GMA and our annual conferences continues to grow, a prolongation of the congress time might be worth considering. 

Otherwise, there is a risk that an increasing parallelization of sessions might give way to conflicts of interest with the participants. In the future, the size of the event already reached will probably go along with growing demands on the organizing faculties with regard to financing, space offer, or participant management. The organization might be simplified by transferring some special tasks to the GMA office or to a consistent congress organizer.

**Figure 3:**
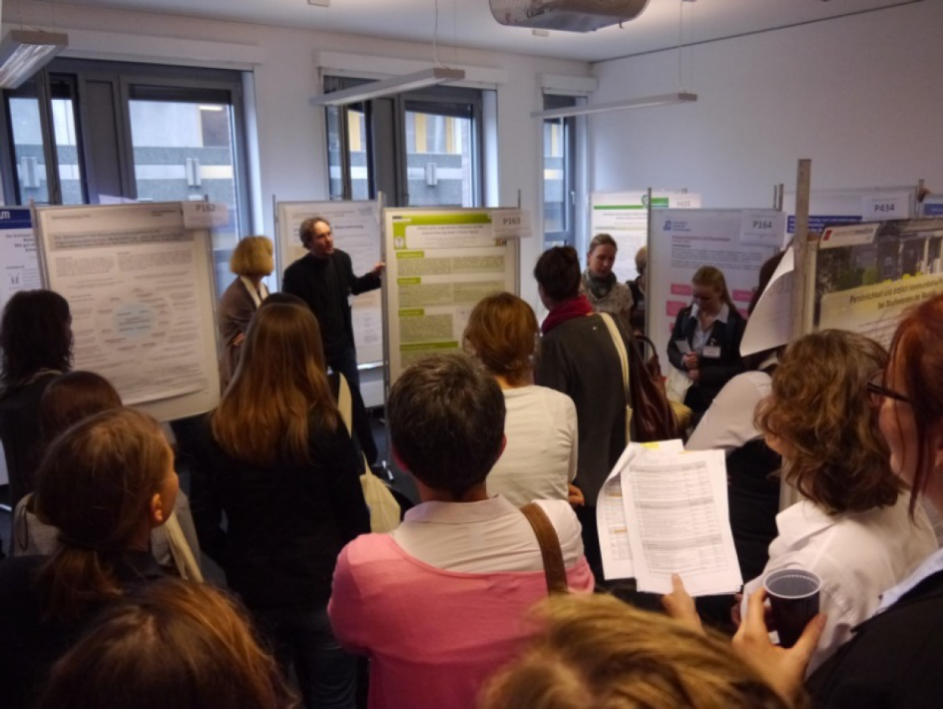
Guided poster tour (photo: Birgit Henkel, UKE)

**Figure 4:**
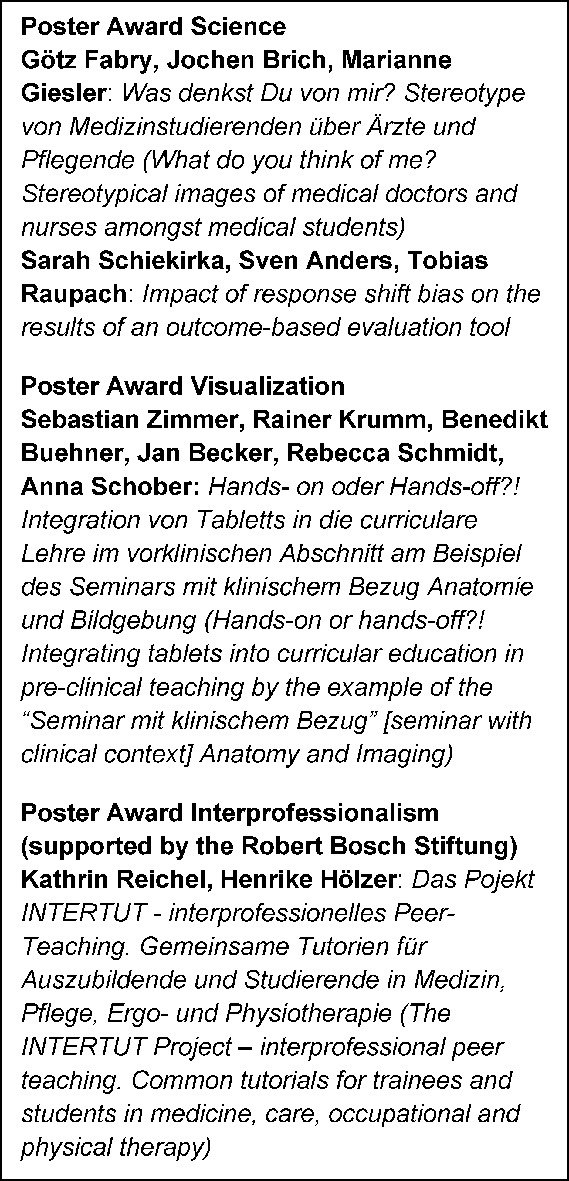
Poster awards

From our point of view, the poster tours (see Figure 3 (Fig. 3)) especially benefitted from the separation of the various contribution types: Many attendees intensively discussed the individual works exhibited in different rooms – and evaluated them for the awarding of the poster prizes. The poster rated best in each poster tour, respectively, made it into the second round, in which an expert panel selected the poster award winners (see Figure 4 (Fig. 4)): Congratulations! The awards and awardees were celebrated during a long poster barbecue night.

Interprofessionalism is a topic with ever growing significance for the GMA. With generous support from the Robert Bosch Stiftung (Robert Bosch Foundation) we were able to promote the cross-curricular and cross-professional integration of education and advanced training in all health professions by means of a workshop, lectures, posters, and a specific poster award (see Figure 4 (Fig. 4)). As part of the board elections during the GMA member assembly, Professor Ursula Walkenhorst was accepted as an advisory board member.

**Figure 5:**
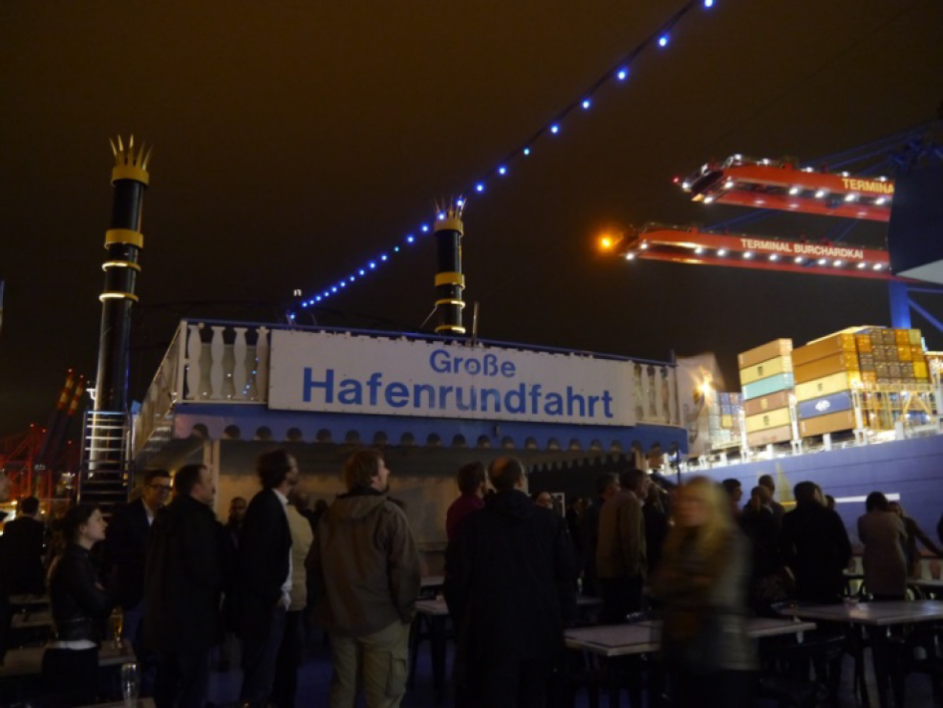
Social evening onboard the paddle steamer “Louisiana Star” (photo: Birgit Henkel, UKE)

**Figure 6:**
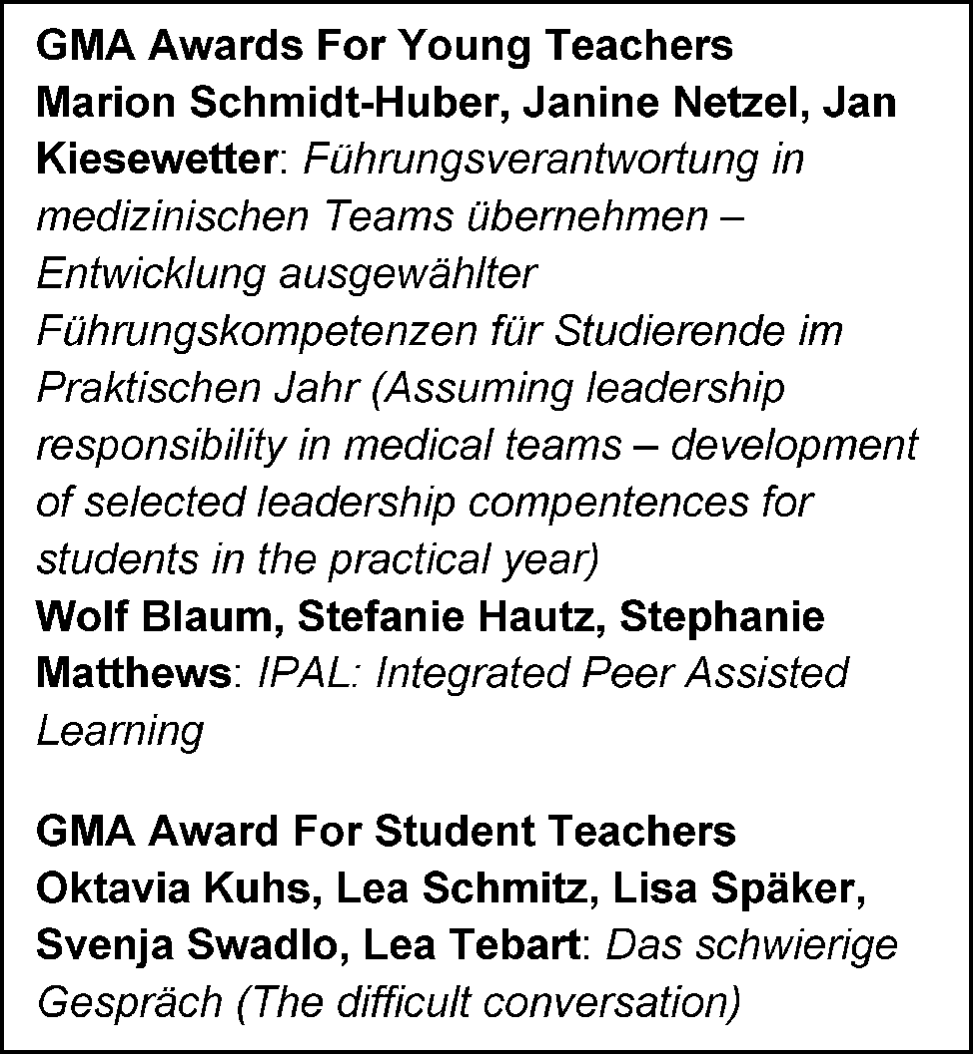
GMA awards

**Figure 7:**
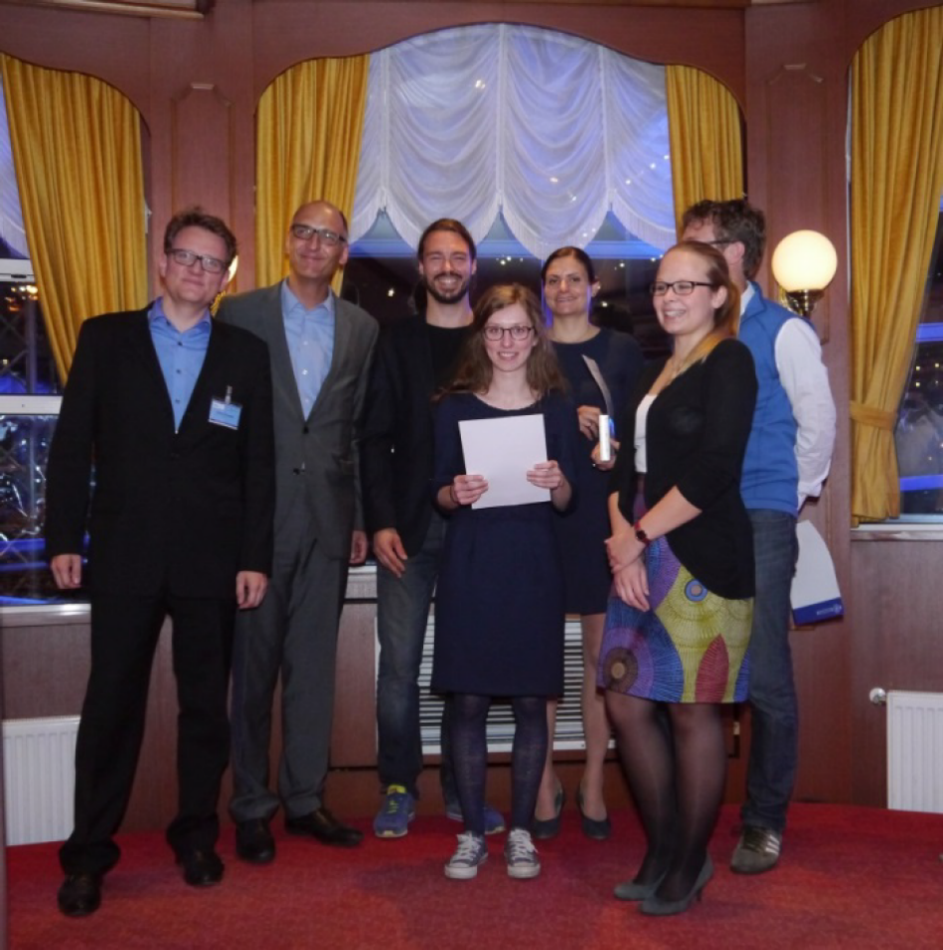
GMA laureates with Sören Huwendiek and Martin Fischer (photo: Birgit Henkel, UKE)

For 390 guests, a very special event was the social evening spent aboard the paddle steamer Louisiana Star, where they enjoyed a maritime buffet accompanied by jazz music (see Figure 5 (Fig. 5)). There was great applause at the presentation of the GMA Awards for Young Teachers and Student Teachers (see Figure 6 (Fig. 6) and Figure 7 (Fig. 7)). 

Afterwards, all landlubbers could witness the nightly activity in Germany’s largest port from the upper deck.

Did you miss any contributions presented at the GMA 2014? Please visit http://www.gma2014.de/programm.html to find the programme and the abstract book. In addition, many participants made use of the first-time offered possibility to upload their presentations or complete posters at https://gesellschaft-medizinische-ausbildung.org/tagungen/2014-hamburg/abstracts.html: Just take a look!

Now, all the positive feedback leaves us wanting more: We wish Rainer Haak and his team many participants and good luck with the next GMA conference to be held in Leipzig, Germany, from September 30th to October 3rd, 2015. The meeting will be fused with the annual conference of the “Arbeitskreis zur Weiterentwicklung der Lehre in der Zahnmedizin” (Working Group for the Advancement of Dental Education), and we look forward to a happy reunion there!

## Competing interests

The authors declare that they have no competing interests.

